# Supplementary material for: Green light enhances the phytochemical preservation of lettuce during postharvest cold storage
Source: PLoS One. 2024 Nov 15;19(11):e0311100. doi: 10.1371/journal.pone.0311100 (PMC11567635; doi:10.1371/journal.pone.0311100)
Supplement: Attachment — Submitted filename: storage manuscript.docx [file pone.0311100.s001.docx]

**Green light enhances the nutritional value and pigment preservation of lettuce during postharvest cold storage**

Shafieh Salehinia^1^, Fardad Didaran^1^, Sasan Aliniaeifard^2^, Saman Zohrabi^1^, Sarah Macpherson^1^, and Mark Lefsrud ^1*^

^1^Department of Bioresource Engineering, Macdonald Campus, McGill University, 21,111 Lakeshore Road, Sainte-Anne-de-Bellevue, Quebec, Canada H9X 3V9

^2^Controlled Environment Agriculture Center, College of Agriculture and Natural Resources, University of Tehran, Tehran, Iran 77871-31587

Corresponding author*: [mark.lefsrud@mcgill.ca](mailto:mark.lefsrud@mcgill.ca)

**Author Contributions**

**Shafieh Salehinia:** Conceptualization, Investigation, Methodology, Formal analysis, Writing-original draft. **Fardad Didaran:** Formal analysis. **Sasan Aliniaeifard:** Methodology, Writing-review & editing. **Saman Zohrabi:** Writing-review & editing**. Sarah MacPherson:** Writing-review & editing. **Mark Lefsrud:** Conceptualization, Methodology, Writing-review & editing, Supervision, Funding acquisition.

# Abstract

The postharvest lighting environment is a main factor that influences quality preservation for harvested biomass. The objective of this study was to evaluate postharvest changes in bioactive compounds of lettuce with different storage light spectra. The effects of green LEDs with peaks at 500 nm and 530 nm, white LEDs (400–700 nm), and dark storage were evaluated, where light intensity (10 µmol m^-2^ s^-1^) and photoperiod (12 h^-d^) were constant with air temperature at 5°C over the 14d treatment period. Lettuce stored with 500 nm and 530 nm green LEDs exhibited 15.7 and 15.5 times higher antioxidant activity, respectively, compared to dark storage. Significant improvements in total phenolic content, and 67.5 % and 64.8 % increases in total soluble solids with 530 nm and 500 nm green LEDs over dark storage were discerned. Exposure to 530 nm green LEDs led to 2.28-fold higher anthocyanin content, a 26.2% increase in carotenoids, and a 95 % rise in flavonoid content compared to dark storage. Increases of 26.4 % and 16.0 % in chlorophyll a content in lettuce stored under 500 nm and 530 nm green LEDs, respectively, and 65.6 % and 46.6 % rises in the Chlorophyll a/b ratio were observed. Compared to dark storage, green LEDs (500 nm) resulted in a 13.5 % higher total chlorophyll content. Findings underscore the positive impact of green LEDs on the nutritional quality of lettuce, providing insight for postharvest practices.

**Keywords:** Antioxidant activity, Bioactive compounds, LED, Light quality, Quality preservation

# Introduction

Lettuce (*Lactuca sativa*) is a widely consumed leafy green on a global scale and it holds significant economic importance. While lettuce is recognized for its high water content and low calorie count, it retains more nutrients when consumed raw compared to other cooked or processed vegetables [1, 2]. This vegetable offers nutritional value, encompassing dietary fibre, minerals, vitamins, antioxidants, and phytochemicals such as carotenoids and phenolic compounds [1, 3, 4]. Lettuce is abundant in cellulose, which plays a crucial role in promoting digestive health and facilitating digestion. Leafy vegetables that have been freshly harvested remain biologically active and continue their metabolic processes post-harvest; in addition, changes in color, chlorophyll degradation, wilting, and decay are some of the factors that affect harvested lettuce, resulting in a decline in its quality and subsequent rejection by consumers [4-7]. It has been reported that both postharvest processing and storage of lettuce can have an impact on overall appearance and nutritional quality [4, 8].

Extending postharvest quality and maintaining the nutritional value of vegetables requires specific storage conditions, commonly including low temperatures to reduce respiration, high humidity to decrease transpiration, and controlled concentrations of carbon dioxide, oxygen, and ethylene to postpone senescence [9]. Refrigeration can enhance postharvest longevity, yet despite adequate cold storage conditions, significant breakdown of chlorophyll, degradation of sugars, and loss of antioxidants can still occur within a short timeframe [10]. Moreover, numerous strategies, including chemical treatments, edible coatings, modified atmosphere packaging (MAP), controlled atmosphere storage, ultrasound, ozone application, and laser irradiation, have been implemented to enhance quality preservation, aiming to reduce quality deterioration and waste [6, 11-18]. However, concerns have been raised regarding cost, effectiveness, practicality, and food safety associated with these conventional methods [19].

Developing an energy-efficient and cost-effective method to prolong the storage life of harvested vegetables while ensuring safety, such as the use of light emitting diodes (LEDs), could potentially address these issues [18, 20-23]. LEDs offer several advantages, including being non-toxic, cost-efficient, and leaving no residue when compared to traditional preservation methods [24-26]. Furthermore, LEDs’ light intensity and spectral properties can be adjusted [27, 28].

Multiple studies have illustrated the effectiveness of LED irradiation in maintaining or enhancing the nutritional attributes of various fruits [29-31], flowers [32, 33], as well as a range of vegetables including lettuce (*Valerianella olitoria*), broccoli (*Brassica oleracea var. italica*), pakchoi (B*rassica rapa* ssp. chinensis), cabbage (*Brassica oleracea var.* capitata), kale (*Brassica oleracea var.* alboglabra), spinach (*Spinacia oleracea*), and basil (*Ocimum basilicum*) [8, 19, 29, 34-40].

While many studies have investigated the impact of LEDs on postharvest storage of vegetables, the emphasis has primarily been on red, blue, and white lights. To the best of our knowledge, little research has explored the effects of postharvest green light on bioactive compounds such as flavonoid and anthocyanin particularly in leafy vegetables like lettuce.

Guo, Zhou [41] found that green LED lighting, at a rate of 25 μmol.m^2^·s^-1^ over a period of 12 days at room temperature, effectively inhibited the accumulation of malondialdehyde. Additionally, it maintained higher chlorophyll content and antioxidant enzyme activities in cabbage. Jin, Yao [42] discovered that broccoli florets stored under green LED light had a higher sulforaphane content compared to those stored in darkness or under fluorescent lighting for 2 days at 25°C. Castillejo, Martínez-Zamora [43] found that green LED increased total glucosinolate content by 34.5% although green LEDs had no impact on the total phenolic content on ready-to-eat broccoli sprouts (*Brassica oleracea var.* italica) during 15 days at 5 °C compared to traditional storage in darkness . These findings suggest that green LED light may influence biosynthetic pathways through pigment photoreceptor proteins, such as cryptochromes and phytochromes (Castillejo et al., 2021a; Meng & Runkle, 2019).

Our study aims to explore the post-harvest exposure of lettuce to green LED light, inducing abiotic stress to stimulate secondary metabolite synthesis. We seek to evaluate the effects of green LED light on key health-promoting compounds in lettuce, conventional storage in darkness or under white LEDs, ultimately benefiting consumers.

# Results

## Effects of postharvest light spectra on antioxidant system of lettuce

The antioxidant activity of the lettuce leaf samples decreased following 14 d of cold storage compared to the initial evaluation (p < 0.01). Remarkably, the reduction in antioxidant activity was less noticeable in lettuce exposed to green LEDs compared to those stored in the dark (Fig 1A). Samples stored in the dark experienced a drastic decrease in antioxidant activity, declining by 94.5 % compared to their initial values. In contrast, light-exposed samples maintained at least 80 % of their antioxidant activity. Specifically, white LED-treated samples showed a reduction of 20.6 %, while samples exposed to 530 nm and 500 nm green LEDs exhibited a 15.7 % and 14.4 % decrease, respectively. Samples exposed to 500 nm, 530 nm, and white light retained respectively 15.7, 15.5, and 14.6 times higher antioxidant activity compared to the antioxidant activity of lettuces stored in the dark at the end of the experiment. Overall, green light exposure prevented a drastic decrease in total antioxidant activity in lettuce leaves during postharvest cold storage.

**Fig 1.** Effect of postharvest light spectra on antioxidant activity (A), total phenolic content (B) and Total soluble solid [TSS; (C)] in lettuce leaf samples stored under different light conditions, including 530 nm and 500 nm green LEDs, white LEDs (400-700 nm), and dark storage for a duration of 14 d at 5 °C. Values represent the means of three replications ± SE. Data in columns with different letters are significantly different p < 0.01.

The total phenolic content of lettuce stored in the dark and under white LEDs decreased significantly to similar levels after 14 d of storage, compared to their initial values (p < 0.01). In contrast, total phenolic content increased significantly in lettuce stored under 530 nm and 500 nm green LEDs compared to their initial values (p < 0.01). Furthermore, green LED-stored lettuce exhibited a significant increase for 530 nm and 500 nm in total phenolic content compared to its content in dark-stored samples (p < 0.01). However, there was no significant difference between white LED- treated samples and those kept in the dark (Fig 1B).

The impact of the experimental LED spectra on the total soluble solid (TSS) content in lettuce is illustrated in Fig 1C. No significant differences were observed between the dark and white treatments and their initial values. However, among the different light treatments, the TSS of lettuce exposed to 530 nm green LEDs demonstrated the highest increase (67.5 % increase), followed by 500 nm green LEDs (64.8 % increase) compared to their initial value and dark storage. These results indicate that green light notably increased the TSS content of lettuce in comparison with the dark storage conditions and their initial values (p < 0.05).

## Effects of postharvest light spectra on protective pigments of lettuce

Total anthocyanin content in all lettuce samples significantly decreased during postharvest storage (p < 0.01). However, the 530 nm green LEDS prevented a considerable decrease in anthocyanin content, exhibiting only a 36.4 % decline compared to its initial value. Anthocyanin content in lettuce stored with 530 nm green LEDs notably contained a higher anthocyanin content (p < 0.01) in comparison to samples exposed to the dark (2.28 times higher). There was no significant difference between the lettuce samples exposed to 500 nm green LEDs and dark storage, showed 72.1 % and 75.2 % decrease compared to their initial values, respectively. However, the samples stored under white light experienced the most drastic decline, with a 91.4 % decrease, in comparison with the initial values and a 69.2 % decline compared to the samples stored in the dark (Fig 2A).

**Fig 2.** Effect of postharvest light spectrum on protective pigments including anthocyanins (A), carotenoids (B), and total flavonoids (C) in lettuce leaf samples stored under different light conditions, including 530 nm and 500 nm green LEDs, white LEDs (400–700 nm), and dark storage for a duration of 14 d at 5 °C. Values are the means of three replications ± SE. Data in columns with different letters are significantly different p < 0.01.

Total carotenoid content of lettuce stored under both the 530 nm and 500 nm green LEDs increased significantly to similar levels compared to their initial values (p <0.01) after 14 d of storage (Fig 2B). This increase was 12.7 % for 530 nm and 11 % for 500 nm in comparison with their initial value, and 26.2 % and 24.2 % in comparison with the dark-stored samples. Conversely, lettuce samples stored with the white LEDs or dark experienced a significant reduction of 8.2 % and 10.7 % in total carotenoid content compared to the initial value (p < 0.01), respectively. There was no significant difference between the carotenoid content of dark and white light-exposed samples.

Lettuces stored under both green LED spectra exhibited a significantly higher total flavonoid content compared to the white light treatment and dark storage. White LEDs had no significant effect on the total flavonoid content of lettuce leaf during 14 d of postharvest storage. Total flavonoid content at d 14 of postharvest storage was increased by 2.6 and 2.2 folds as a result of lettuce leaf exposure to 530 nm and 500 nm green lights, respectively (Fig 2C). The samples stored in the dark had a 1.3-fold increase in the total flavonoid content. Furthermore, 530 nm and 500 nm exposure caused respectively 95 % and 65 % increase in flavonoid content compared to its content in the samples stored in the dark, whereas the white LED treatment caused 25.96 % decrease compared to the flavonoid content of dark-stored samples following 14 d of postharvest storage.

## Effects of postharvest light spectra on photosynthetic pigments in lettuce

Chlorophyll *a* content decreased significantly in both dark and white light-exposed samples during storage (p < 0.01). Interestingly, lettuce stored with green 500 nm LEDs exhibited a notably higher chlorophyll a content compared to the initial value (11.3 %). Compared to the initial value, the chlorophyll *a* content in samples stored in the dark and under white LEDs decreased by 12 % and 9.3 %, respectively. Conversely, there was no changes between those stored under the 530 nm green LEDs and their initial value. Lettuce leaf samples exposed to 500 nm and 530 nm green LEDs displayed respectively 26.4 % and 16.0 % higher chlorophyll *a* content compared to samples stored in dark, while the white LED treatment showed no significant difference with chlorophyll *a* content in dark-exposed samples (Fi 3A).

**Fig 3.** Effect of postharvest light spectrum on photosynthetic pigments including chlorophyll (Chl) *a* (A), Chl *b* (B), Chl *a*/*b* ratio (C), and total Chl content in lettuce leaf samples stored under different light conditions, including 530 nm and 500 nm green LEDs, white LEDs (400-700 nm), and dark storage for a duration of 14 d at 5 °C. Values are the means of three replications ± SE. Data in columns with different letters are significantly different (p < 0.01).

All lettuce samples maintained a statistically similar level of chlorophyll *b* content, except for those exposed to 530 nm green light, where the chlorophyll *b* content decreased by 29 % compared to both their initial values or for the dark storage (p < 0.05). In the case of the 500 nm green LEDs, there was a 10 % reduction in the chlorophyll *b* content compared to their initial value for the dark storage; however, this reduction was not significantly different (Fig 3B).

The Chl *a*/*b* ratio in lettuce leaf samples treated with dark or white LEDs exhibited 13.2 % and 10.0 % decreases, respectively, in comparison with their initial ratio (Fig 3C). However, these reductions were not statistically significant compared to the initial values. Conversely, lettuce treated with 530 nm and 500 nm green LEDs showed 43.6 % and 27.1 % increases, respectively, in Chl *a*/*b* ratio in comparison to their initial ratios (p < 0.01). Compared to the dark condition, the 530 nm and 500 nm LEDs had a 65.6 % and 46.6 % increase in Chl *a*/*b* ratio (p < 0.01), respectively, yet there was no significant difference between these two green LEDs. In contrast, the white LED and dark treatments showed no significant differences with each other or with their initial ratios.

Compared to the initial content, lettuce samples exhibited a notable reduction in total chlorophyll content (7.7 %, 7.6 %, and 6.1 % for 530 nm, dark storage, and white LED treatments, respectively), with the exception of those stored under 500 nm green light (Fig 3D). In the case of the 500 nm green LEDs, a significant increase (4.8 %) in total chlorophyll content was detected compared to the initial content (p < 0.01). There was no significant difference between the total chlorophyll content in samples treated with white and 530 nm green LEDs and those stored in the dark. In contrast, the 500 nm green LEDs led to a 13.5 % higher total chlorophyll content compared to samples stored in dark (p < 0.01).

## Principle component and correlation matrix analyses for the bioactive compounds and color of lettuce as affected by postharvest lighting environment

A matrix correlation analysis was conducted to examine the relationship between bioactive compounds and color attributes in the lettuce leaf samples stored under different light conditions, including green LEDs with peaks at 500 nm and 530 nm, white LEDs, and dark storage. The Pearson correlation coefficients exhibited intricate linkages among the variables under investigation (Fig 4).

**Fig 4.** The correlation matrix for the effect of postharvest light spectrum on bioactive compounds of lettuces. The samples were exposed to 530 nm and 500 nm green light, white light (400-700 nm), and dark for a duration of 14 d at 5 °C.

The principal component analysis (PCA) revealed information regarding the levels of bioactive chemicals and color characteristics in lettuce following 14-d storage period at 5°C, while being subjected to different light treatments (Fig 5). The PCA biplot exhibits a notable clustering pattern, indicating a clear differentiation among treatments. Notably, the treatments involving green LEDs with peak wavelengths at 500 nm and 530 nm exhibit a prominent separation. The graph provides a visual representation of the key components that contribute to the observed changes. The results of the subsequent clustering analysis corroborate and extend these findings by explicitly delineating sample groupings based on similarity. Under dark storage conditions, both at initiation (d 0) and end of the storage period (d14), samples formed distinct clusters (Cluster 1 and Cluster 2, respectively), indicating a shift in biochemical composition over time. Similarly, samples exposed to white LEDs exhibited cluster transitions from d 0 to d 14, further elucidating the evolving nature of the lettuce characteristics. Of particular interest are the samples treated with green LEDs at 500 nm and 530 nm. At d 0, leaf samples stored under both 500 nm and 530 nm green LEDs aligned with Cluster 1, signifying a certain degree of similarity with the dark and white conditions. However, by d 14, a notable divergence occurred, with 500 nm green LEDs at d 14 and 530 nm green LEDs at d 14 forming a distinct Cluster 3. This divergence, highlighted by increased distances to centroids, underscores the profound impact of these specific light treatments on the biochemical profiles of the lettuce during the storage period.

**Fig 5.** Principle component analysis (PCA) for the effect of postharvest light spectrum on bioactive compounds of lettuces. The samples were exposed to 530 nm and 500 nm green light, white light (400-700 nm), and dark for a duration of 14 d at 5 °C.

# Discussion

This study aimed to explore the impact of green LEDs, white LEDs and dark storage on the maintenance of nutritional value in lettuce. Levels of chlorophylls, carotenoids, flavonoids, antioxidants, anthocyanin, phenolic compounds, soluble solids, and color characteristics were compared in lettuce both before and after the 14-d storage period.

## Antioxidant capacity

LED lighting has proven a valuable tool for preserving phenolic compounds and antioxidant activity in harvested produce [44, 45]. Our research indicates that employing green LEDs (specifically with a peak at 530 nm) effectively increased total phenolic content, thereby leading to a significant retention of total antioxidant activity in lettuces, as compared to dark storage and other light treatments. Similar outcomes have been reported by Lee, Ha [37], where cabbage stored with green LED irradiation led to an increase in the total polyphenol content and total antioxidant activity in comparison to dark storage, aligning with findings reported by Jin, Yao [42] for broccoli and Samuolienė, Brazaitytė [46], Samuolienė, Sirtautas [47] for baby leaf lettuce. This phenomenon is likely attributed to the induction of phenylalanine ammonia-lyase activity, a crucial enzyme in the phenylpropanoid pathway, in lettuce exposed to green light [48]. Higher retention of antioxidant activity in green LED-treated lettuce at 530 nm in our work could be mainly ascribed to their higher levels of total phenolics, flavonoids or anthocyanins in comparison to dark storage, which is in accordance with Samuolienė, Brazaitytė [46]. It has been suggested that combining different antioxidants can create synergistic effects, potentially enhancing their overall antioxidant capacity [49]. Further investigation and experimentation, including molecular and physiological studies, could provide a more detailed understanding of the mechanisms underlying these exceptions.

Kasim and Kasim [4] observed that green light significantly contributed to the preservation of chlorophyll content and TSS in lettuce when compared to a dark environment after a 21-d storage. Kim, Lee [50] indicated a gradual increase in TSS in light-treated groups, surpassing the levels observed in the dark for strawberries after 4-d storage. According to Zhan, Hu [51] illuminating fresh broccoli with light during the initial storage phase conserved or stimulated the production of photosynthetic products. The outcomes of the current study regarding lettuce's TSS align with these previous findings.

## Protective pigments

Data presented herein show that 530 nm green LEDs resulted in a significant decline in anthocyanin content compared to dark storage, emphasizing its potential significance in preserving these vital compounds. Although the effect of green light at 500 nm was relatively modest, showing similar anthocyanin content to dark conditions, it still presented a positive influence when compared to white light. Red light exposure during growth significantly boosts the anthocyanin content in lettuce compared to white light treatment [52] and green and blue LEDs tend to enhance anthocyanin content in herbs [49, 53]. Gitelson, Merzlyak and Chivkunova [54] observed that the absorbance of maple (*Acer platanoides*), cotoneaster (*Cotoneaster alaunica*) leaves in the vicinity of 550 nm and within the 400–600 nm band is directly proportional to the anthocyanin content. An increase in anthocyanin levels results in heightened absorbance within the green spectral range around 550 nm [47]. The implications of these findings underscore the intricate relationship between light quality and the preservation of essential compounds such as anthocyanins in plants, highlighting potential pathways for optimizing their accumulation through various metabolic responses [55, 56].

## Postharvest green light exposure increased total carotenoid content in lettuce leaves

Research has indicated that chlorophyll and carotenoid content tends to diminish during storage [57]. Light quality plays a crucial role in determining carotenoid accumulation [58]. For instance, exposure to red light leads to elevated lycopene content in tomatoes and for broccoli, exposure to blue light at 20 μmol m^−2^ s^−1^ at 22 °C for 3 d resulted in increased carotenoid levels [59]. Also, lamb lettuce exposed to warm white light exhibited a slower decline in carotenoid content [8]. However, Loi, Liuzzi [19] showed no significant difference between green (524 nm), blue (467 nm), red (625 nm) and yellow (587 nm) at an intensity of 24 μmol m^−2^ s^−1^ and dark control after 20 d of storage of broccoli. In our work, the green LED-treated samples exhibited a greater quantity of total carotenoids compared to the dark control samples (26% and 24% for 530 and 500 nm, respectively), their initial value or white LED-treated samples. This increase in pigment content may be attributed to the positive influence of green light on the synthesis of carotenoids and chlorophylls [60]. Consistent with our results, Pennisi, Orsini [44] was found that the carotenoid content remained higher (21 % higher in comparison with dark-stored samples) after 10 d of storage in red chards stored under 517 nm green light with an intensity of 35 μmol m^−2^ s^−1^. In our experiment, a low light intensity (10 μmol m^−2^ s^−1^) was used, which could explain the higher pigment content after 14 d of exposure to green LEDs light. Hasperué, Rodoni [60] observed a positive effect with white-blue LEDs on postharvest carotenoid accumulation in Brussel sprouts (*Brassica oleracea* var gemmifera).

## Total flavonoid content enhanced by green light during postharvest storage

The total flavonoid content in lettuce stored under green LEDs was significantly higher compared to lettuce stored in the dark or under white light, in our study. The increased content of flavonoids in the green-treated samples may be attributable to the light quality. Previous studies have shown similar results in which the flavonoid synthesis stimulated by exposure to light as a protective response [60-62]. Alterations in reactive oxygen species (ROS) stimulate the biosynthesis of flavonoids, which have the capability to scavenge singlet oxygen and stabilize chloroplast membranes [60, 63]. Kanazawa, Hashimoto [61] tested UV-A, UV-B lamps and green LEDs on parsley (*Petroselinum crispum*), Indian spinach (*Basella rubra*) and garden pea sprout (*Pisum stivum*) storage in a home refrigerator. They demonstrated that light with wavelengths near the absorption λmax of phenylpropanoids, particularly around 503 nm for pelargonidin glycoside, can induce flavonoid synthesis. Additionally, the expression of genes involved in phenylpropanoid biosynthesis was found to induce flavonoid synthesis [61, 64]. For vegetables with anthocyanin content and a λmax around 503 nm, green light in the 500−600 nm range is effective in inducing flavonoid synthesis.

## Photosynthetic pigments

Senescence in harvested green organs typically involves the extensive breakdown of chlorophylls and chloroplast proteins [26, 44, 65]. These responses are influenced by the quality and intensity of light [66]. Excessive light intensity can lead to photo-oxidative damage, resulting in the degradation of chlorophylls and carotenoids [44, 58]; therefore, in this study a low level light (10 μmol m^−2^ s^−1^) was used to prevent photo-oxidative damages to the leaves [67]. Under the experimental conditions employed, total chlorophyll content of all lettuce samples exhibited a decrease, with the exception of those subjected to storage under green (500 nm) LEDs, which displayed a notable augmentation in total chlorophyll content. The observed increase in chlorophyll content and intensified green coloration in the lettuce following 14-d cold storage is noteworthy and consistent with consumer preferences for high-quality produce. The increased concentration of pigments can be ascribed to the beneficial impact of green light, particularly at a wavelength of 500 nm, on the process of chlorophyll synthesis and the promotion of chlorophyll metabolism [68]. This effect is influenced by the reduced electron transport and/or the heightened abundance of divinyl chlorophyllide *a*, a significant precursor in the biosynthesis of chlorophyll [69]. The potential influence of low-level light on chlorophyll-related genes and enzymes during post-harvest handling has been investigated by several researchers [69-71]. According to Zhou, Zuo [70], the upregulation of the BrHEMA1 gene, which is involved in chlorophyll synthesis, is observed in samples treated with LED light. This upregulation leads to an increase in chlorophyll production and a decrease in the expression of genes responsible for chlorophyll breakdown. Nevertheless, it is plausible that there exist alternative explanations for the observed augmentation in chlorophyll levels in lettuce exposed to green (500 nm) LED lighting following a 14-d storage period.

## Postharvest green light (500 nm) exposure enhanced the Chl a content in lettuce

The observed rise in Chl *a* concentration following postharvest exposure to 500 nm light can be attributed to the lettuce leaves' adaptive responses aimed at maximizing photosynthetic efficiency. This reaction may encompass the metabolic processes that promote the synthesis of Chl *a* and the genetic variety present in the plant [72]. In contrast, the observed reduction in Chl *b* concentration subsequent to exposure to 530 nm light may be attributed to the preferential absorption characteristics of Chl *b*. Chl *b* predominantly absorbs light within the blue and red-orange regions of the electromagnetic spectrum, while exhibiting lower efficiency in absorbing green light (about 530 nm), and absorption of Chl *b* may be negatively affected by exposure to the 530 nm wavelengths. Another potential factor contributing to this phenomenon could be modifications in chlorophyll *a* to chlorophyll *b* ratio, which serves to enhance the efficiency of photosynthetic processes. It is worth noting that a significant increase in the Chl a/b ratio of lettuce was observed after storage under green LEDs, particularly with a wavelength of 530 nm, compared to lettuce stored under white LEDs or in the dark. This implies that the utilization of green LED light could potentially improve the postharvest storage and preservation of an ideal Chl *a*/*b* ratio, boosting light absorption and the efficiency of photosynthesis. In the context of postharvest settings, it is often preferable for the Chl *a*/*b* ratio to have a larger value, since this signifies a prevalence of Chl *a*. This particular form of chlorophyll has been observed to give more favorable outcomes. Furthermore, previous studies have provided evidence that plants have the ability to modify their chlorophyll concentrations in response to variations in light intensity and quality [8]. This adaptation results in an elevated ratio of Chl *a*/*b* ratio to optimize the capture of red light [73], and in this way affecting both leaf light absorption and visual quality. Alternatively, in situations with limited light availability, the Chl *a*/*b* ratio may be decreased to improve the absorption of blue light [74, 75]. According to Lee, Ha [37], the distribution of chlorophyll inside plants leads to variations in the absorption of light wavelengths. Additional empirical research is required in order to validate these mechanisms and delve into their implications within the context of lettuce preservation. Previous research indicates that the use of green light has a beneficial effect on the chlorophyll content of postharvest vegetables. For instance, the application of green LED light has been found to either preserve or boost the chlorophyll content in broccoli heads (*Brassica oleracea* ssp. italica) [19, 42]. Continuous postharvest illumination with green light results in elevated overall chlorophyll content in cabbage (*Brassica oleracea* var. dongdori) [37], as well as preserving of chlorophyll content in rocket (*Diplotaxis tenuifolia*) [44].

## Relationship between preserving different bioactive compounds in lettuce

PCA and correlation matrix analyses performed in this work offer new insight into the complex biochemical dynamics of lettuce subjected to different light spectra.

It is worth mentioning that there was a strong positive association between the chlorophyll *a*/*b* ratio and the amount of flavonoids and phenolics. This suggests that there may be connections between the dynamics of pigmentation and the accumulation of secondary metabolites. The statistical significance of the observed connections is supported by the confidence intervals associated with these correlations, thus enhancing the reliability of the findings. The utilization of graphical depictions to illustrate these correlations has the potential to augment the visual understanding of these complex associations, hence promoting a more nuanced interpretation among members of the scientific community.

A strong negative correlation between chlorophyll *a* and chlorophyll *b* highlights the synchronized regulation of vital photosynthetic pigments that are crucial for optimizing the process of photosynthesis. In addition, the observed strong correlation between the chlorophyll *a*/*b* ratio and flavonoids and phenolics implies a potential interaction between pigmentation and the formation of secondary metabolites.

Green LEDs appear advantageous for chlorophyll content, total flavonoids, and phenolic content, adding to the extensive network of linkages that exist within lettuce's biochemical responses to varying light conditions [61, 76, 77]. These findings underscore the potential benefits of utilizing green 500 nm and 530 nm LED light as a tool to maintain and improve the nutritional composition of stored lettuce, driving advancement of post-harvest storage systems, with the potential to bring about changes in the practices within the agricultural and food industries. These data support our previous study, which showed the effectiveness of green wavelengths in keeping stomata in a closed state in both basil (*Ocimum basilicum*) and lettuce, suggesting its potential in maintaining postharvest quality and prolonging the shelf life of leafy vegetables [23].

# Conclusion and future perspectives

Exposure of lettuce to light throughout its storage period can yield advantageous outcomes for a duration of 14 d at 5 °C. Noteworthy findings include a significant increase in antioxidant activity, where samples exposed to 500 nm and 530 nm green LEDs displayed 15.7 and 15.5 times higher activity, respectively, compared to those stored in the dark. Substantial enhancements in total phenolic content were additionally observed, and green 530 nm and 500 nm treatments resulted in 67.5 % and 64.8 % increases in total soluble solids compared to dark storage. Exposure to 530 nm green light further led to a 2.28-fold higher anthocyanin content, a 26.2 % increase in carotenoids, and a 95% rise in flavonoid content compared to dark-stored samples. Chlorophyll-related parameters showed increases of 26.4 % and 16.00 % in chlorophyll a content for 500 nm and 530 nm, respectively, along with a 65.6 % and 46.6 % rise in the Chl a/b ratio. The 500 nm treatment resulted in a 13.5% higher total chlorophyll content compared to dark-stored samples. More precise examination of light spectra, a diverse range of green LEDs, and potential benefits from combining modified environmental packaging with suitable storage conditions for various vegetable varieties should be explored. Future studies could further focus on finely adjusting combinations of light spectral composition, experimenting with various green LEDs, and exploring light integrals to specifically target improvements in the quality and storability of different vegetables, or investigating different combinations of light with modified atmosphere packaging.

# Material and methods

## Plant material

Romaine lettuce (*Lactuca sativa* var. Breen) was harvested from La Boîte Maraîchère (LBM) a local commercial vertical hydroponic farm located in Laval, Quebec, Canada. The lettuce was promptly transported to the laboratory within one hour of harvesting. Fresh lettuce heads, exhibiting uniform size and color, and showing no signs of disease or mechanical damage, were selected for the experiment.

## LED lighting

Two different green LED assemblies with peak wavelengths at 500 nm and 530 nm, along with white LEDs (UTechnology, Calgary, Alberta, Canada) were used for the experiment (Fig 6). The photosynthetic photon flux density (PPFD) necessary for post-harvest preservation is typically minimal, spanning from 10 to 80 μmol m^−2^ s^−1^ as indicated in multiple research studies [78]. The light intensity employed in this experiment was set at 10 µmol m^-2^ s^-1^ PPFD, based on our preliminary research (data not shown). This intensity level was measured with a quantum meter (Model MQ500, Apogee Instruments Inc., Logan, UT, US). A consistent 12-h photoperiod was maintained to simulate the lighting conditions in retail stores.

**Fig 6.** Spectral distribution and peaks of three LED assemblies used in this study including green LEDs with peaks at 500 nm and 530 nm, as well as white LEDs (400–700 nm). LED: light emitting diode.

## Packaging and storage conditions

Lettuce heads were randomly divided into four experimental groups, each consisting of three lettuces. Each individual lettuce head was placed within a transparent clamshell polyethylene terephthalate (PET) container (LBM AGTECH, Laval, QC, CA) measuring 30 cm (l) x 13 cm (w) x 15 cm (h). These groups were allocated to different shelves (dimensions: length 66 cm, width 32 cm, and height 27 cm), each outfitted with LEDs emitting distinct wavelengths (Fig 7). The shelves were shielded with black plastic to prevent light transmission to the other samples. The entire setup was situated in a cold storage room and maintained for a period of 14 d. A control group was included in the experiment and subjected to dark storage conditions. Throughout the duration of the experiment, the cold storage room was maintained at 5 °C ±1 °C and a relative humidity (RH) ranging from 90 % to 95 %. Temperature and humidity data were collected at five-minute intervals using a data logger (Elitech RC51H, Elitech Technology, San Jose, CA, US) installed within each shelf. The entire experiment was conducted in triplicate.

**Fig 7.** Representative image of lettuces under different cold storage conditions, including dark storage (top left shelf), green LEDs with peaks at 500 nm (top right shelf) and 530 nm (bottom right shelf), and white LEDs (400–700 nm; bottom left shelf).

## Extract preparation

Three lettuce head samples were collected on d 0 (initial day) and freeze-dried using a laboratory-scale vacuum freeze-drier (Martin Christ Gefriertrocknungsanlagen GmbH Gamma 1-16 LSCplus, Osterode, Lower Saxony, Germany). Freeze- drying was carried out at room temperature for 48 h. After 14 d in cold storage, representative lettuce samples from each experimental light group and dark storage were collected. All freeze-dried samples were subsequently stored in a plastic bag in a dark space at room temperature (20 °C). For extract preparation, 1 g freeze-dried lettuce sample was immersed in liquid nitrogen before grinding with a mortar and pestle. After 5 min (when there was < 1 mg change in mass), samples were transferred to 50-ml Falcon tubes and 30 mL absolute methanol (Thermo Fisher Scientific, Waltham, MA, US) was added. Falcon tubes were vortexed (Thermo Scientific vortex, Waltham, MA, US) for 20 min at 40 g. The extracts were incubated in the freezer at -20 °C in the dark for 48 h. After 2 d, each sample was vacuum-filtered using Whatman TM filter paper (Thermo Fisher Scientific, Waltham, MA, US). The extracts were further diluted with methanol (1:6 ratio or 2 mL extract+10 mL methanol) and subjected to analysis.

## Total chlorophyl and carotenoid content

The lettuce extracts subjected to analysis using a UV/visible spectrophotometer (Ultrospec 2100 pro, Biochrom Limited, Cambridge, England), at wavelengths of 470 nm, 652.4 nm, and 665.2 nm. Total chlorophyll a (Chl a), chlorophyll b (Chl b), and carotenoid content were determined with Equations 1, 2, and 3 below, outlined previously by Lichtenthaler and Buschmann [79]:

$\mathrm{Chl}a = 16.72* \mathrm{Abs}_{665.2}- 9.16 {*Abs}_{652.4}$	(1)

$\mathrm{Chl}b =34.09*\mathrm{Abs}_{652.4}-15.28*\mathrm{Abs}_{665.2}$	(2)

$Carotenoid =(1000* \mathrm{Abs}_{470}-1.63*\mathrm{Chl}_{a}-104.96*\mathrm{Chl}_{b})$	(3)

Results are expressed on a dry mass basis. All tests and analyses were conducted in three replicates.

## Total anthocyanin content

Total anthocyanin content in the lettuce samples was determined using a pH differential method [80]. Briefly, a pH 1.0 buffer reagent was prepared by mixing 980 mL distilled water with 1.86 g KCl in a 1-L beaker. HCl was then used to modify the pH to 1.0 ± 0.05. The volume was brought up to 1L with distilled water in a 1-L volumetric flask. A reagent with a pH of 4.5 was prepared in a beaker by combining 54.43 g sodium acetate trihydrate (CH_3_CO_2_Na·3H_2_O) with distilled water to a volume of 960 mL. Following the addition of HCl to modify the pH to 4.5 ± 0.05, the buffer was brought up to 1 L with distilled water in another 1-L volumetric flask. The lettuce extracts were diluted by combining the 2 mL extract with 10 mL of absolute methanol. Subsequently, two aliquots were prepared for each diluted extract: one containing 1 mL of diluted extract in 4 mL of a pH 1.0 buffer solution, and the other containing 1 mL of diluted extract in 4 mL of a pH 4.5 buffer solution. For the determination of the total anthocyanin content, the absorbance of the test portion diluted with pH 1.0 and pH 4.5 buffers was measured at 510 and 700 nm, respectively, using a plate reader (BioTek Synergy HTX Multimode Reader, Agilent Technologies, California, USA) and Equation 4:

$Anthocyanin pigment content=(A*MW*DF*1000)/\varepsilon$	(4)

Where, $A=\left( \mathrm{Abs}510 - Abs700 \right)pH 1 - \left( \mathrm{Abs}510 - Abs700 \right)pH 4.5$

$\mathrm{MW}$ (cyanidin – 3 – glucoside) = 449.2_,_ $\mathrm{DF}$ = dilution factor_,_ $\varepsilon$(Molar absorptivity) = 26, 900_._ Results were expressed as mg of cyanidin-3-glucoside equivalent per g of dry mass. All tests and analyses were conducted in three replicates.

## Total phenolic content

Total phenolic content (TPC) was assessed employing the Folin-Ciocalteu reagent (FCR) protocol [81]. Initially, the FCR was diluted ten-fold with distilled water (1 mL FCR (Sigma-Aldrich, Saint Louis, MI, US) and 9 mL distilled water). Next, 10 μL of each sample extract was combined with 75 μL diluted FCR solution, and this mixture was allowed to incubate at room temperature for 10 min. Following this incubation, 75 μL of 2 % sodium carbonate (Na_2_CO_3_) solution (Sigma-Aldrich, Saint Louis, MI, US) was added, and the resulting mixture was vigorously vortexed for 15 seconds. It was left to incubate for 45 minutes in the absence of light. A blank solution consisting of 70% methanol was used for reference. The absorbance of each extract was then measured at a wavelength of 765 nm using a plate reader (BioTek Synergy HTX Multimode Reader, Agilent Technology, CA, US). To generate a standard curve, gallic acid was used at five different concentrations ranging from 0.01 to 0.08 g L^-1^. The total phenolic content of the lettuce extracts was expressed as g of gallic acid equivalents per g of dry extract mass (g GAE g^-1^). All tests and analyses were conducted in three replicates.

## Total flavonoid content

Total flavonoid content (TFC) of lettuce was quantified utilizing the aluminum chloride colorimetric method [81]. Specifically, 15 μL of each lettuce sample extract was combined with a mixture consisting of 45 μL of 70 % methanol (Thermo Fisher Scientific, Waltham, MA, USA), 3 μL of 10 % aluminum chloride hexahydrate (Sigma-Aldrich, Saint Louis, MI, USA), 3 μL of 1 M sodium acetate (Sigma-Aldrich, Saint Louis, MI, USA), and 84 μL of distilled water. Subsequently, this mixture was incubated for a duration of 40 minutes. Following incubation, the absorbance at a wavelength of 415 nm was recorded using a plate reader (BioTek Synergy HTX Multimode Reader, Agilent Technologies, California, US) and used for the determination of the total flavonoid content in duplicate measurements. To generate a standard curve, quercetin was used at concentrations ranging from 0.05 to 0.25 g L^-1^. Total flavonoid content of the lettuce extract was expressed as g quercetin equivalents per g of dry mass (g QE g^-1^).

## Antioxidant activity

The evaluation of antioxidant activity in lettuce was performed using the DPPH method as described previously [82] with some modifications. Briefly, 0.5 mL of diluted extract was introduced into a 1 mL methanolic solution containing DPPH radicals (0.2 mM). The mixture was vigorously vortexed (15 s) and allowed to stand for a duration of 30 minutes. The absorbance was measured at 515 nm using a plate reader (BioTek Synergy HTX Multimode Reader, Agilent Technologies, California, USA). Ascorbic acid (50mM) was employed at five different concentrations ranging from 0.01 to 0.32 g L^-1^. All tests and analyses were conducted in three replicates.

## Total soluble solids

Total soluble solids (TSS) analyses of lettuce leaves were performed using a digital refractometer (ATAGO, model PR32a, Tokyo, Japan) as previously described [83]. A 5 g sample of lettuce leaves was completely ground, followed by centrifugation at 1372 g for 10 min to collect a liquid supernatant. A small quantity of this liquid was used for analysis, placed onto the inspection mirror of the digital refractometer. The refractometer's scale reading (%) was then recorded. All tests and analyses were conducted in three replicates.

## Statistical analysis

Statistical analyses were performed using SAS statistical analysis software developed by SAS Institute Inc. (Cary, NC, US). The experimental design employed a split-plot arrangement within a completely randomized design. The experiment repeated three times. The results given in this study are shown as the mean ± standard deviation (SE). In order to ascertain significant disparities between means, Duncan tests, which were safeguarded by ANOVA, were utilized at a significance level of p < 0.01.

# Acknowledgments

The authors would like to extend their heartfelt appreciation to all their colleagues in the Biomass Production Laboratory for their unwavering support throughout the project, with a special mention of Mr. Yvan Gariepy for his invaluable assistance.

# References

1.	Martínez-Ispizua E, Calatayud Á, Marsal JI, Cannata C, Basile F, Abdelkhalik A, et al. The nutritional quality potential of microgreens, baby leaves, and adult lettuce: an underexploited nutraceutical source. Foods. 2022; (11) 423. doi: <https://doi.org/10.3390/foods11030423>.

2.	Kim MJ, Moon Y, Tou JC, Mou B, Waterland NL. Nutritional value, bioactive compounds and health benefits of lettuce (Lactuca sativa L.). Journal of Food Composition and Analysis. 2016; (49) 19-34. doi: <https://doi.org/10.1016/j.jfca.2016.03.004>.

3.	Abdelgawad KF, Mhmoud AA, Mohamed HF. Foliar spraying with some biostimulants improves growth, chemical constituents, and yield of head lettuce plant. Middle East Journal of Agriculture Research. 2018; (7) 1268-77.

4.	Kasim MU, Kasim R. While continuous white LED lighting increases chlorophyll content (SPAD), green LED light reduces the infection rate of lettuce during storage and shelf‐life conditions. Journal of Food Processing and Preservation. 2017; (41) e13266. doi: <https://doi.org/10.1111/jfpp.13266>.

5.	Barbosa-Cánovas GV. Handling and preservation of fruits and vegetables by combined methods for rural areas: technical manual Food & Agriculture Org.; 2003.

6.	Jiang A, Zuo J, Zheng Q, Guo L, Gao L, Zhao S, et al. Red LED irradiation maintains the postharvest quality of broccoli by elevating antioxidant enzyme activity and reducing the expression of senescence-related genes. Scientia Horticulturae. 2019; (251) 73-9. doi: <https://doi.org/10.1016/j.scienta.2019.03.016>.

7.	Jiang H, Zhang M, Bhandari B, Adhikari B. Application of electronic tongue for fresh foods quality evaluation: A review. Food Reviews International. 2018; (34) 746-69. doi: <https://doi.org/10.1080/87559129.2018.1424184>.

8.	Braidot E, Petrussa E, Peresson C, Patui S, Bertolini A, Tubaro F, et al. Low-intensity light cycles improve the quality of lamb's lettuce (Valerianella olitoria [L.] Pollich) during storage at low temperature. Postharvest biology and technology. 2014; (90) 15-23. doi: <https://doi.org/10.1016/j.postharvbio.2013.12.003>.

9.	Singh V, Hedayetullah M, Zaman P, Meher J. Postharvest technology of fruits and vegetables: an overview. Journal of Postharvest Technology. 2014; (2) 124-35. doi.

10.	Pintos F, Rodoni L, Patrignani M, Ixtaina P, Vicente A, Martínez G, Hasperué J. Advances in the use of white light on broccoli and kale postharvest shelf life. Innovative Food Science & Emerging Technologies. 2023; (86) 103373. doi: <https://doi.org/10.1016/j.ifset.2023.103373>.

11.	Alexandre EM, Brandão TR, Silva CL. Efficacy of non-thermal technologies and sanitizer solutions on microbial load reduction and quality retention of strawberries. Journal of Food Engineering. 2012; (108) 417-26. doi: <https://doi.org/10.1016/j.jfoodeng.2011.09.002>.

12.	Ali LM, Saleh SS, Ahmed AE-RAE-R, Hasan HE-S, Suliman AE-RE. Novel postharvest management using laser irradiation to maintain the quality of strawberry. Journal of Food Measurement and Characterization. 2020; (14) 3615-24. doi: <https://doi.org/10.1007/s11694-020-00600-3>.

13.	An J, Zhang M, Lu Q. Changes in some quality indexes in fresh-cut green asparagus pretreated with aqueous ozone and subsequent modified atmosphere packaging. Journal of Food Engineering. 2007; (78) 340-4. doi: <https://doi.org/10.1016/j.jfoodeng.2005.10.001>.

14.	Chen F, Zhang M, Yang C-h. Application of ultrasound technology in processing of ready-to-eat fresh food: A review. Ultrasonics sonochemistry. 2020; (63) 104953. doi: <https://doi.org/10.1016/j.ultsonch.2019.104953>.

15.	Kerch G. Chitosan films and coatings prevent losses of fresh fruit nutritional quality: A review. Trends in Food Science & Technology. 2015; (46) 159-66. doi: <https://doi.org/10.1016/j.tifs.2015.10.010>.

16.	Shezi S, Magwaza LS, Mditshwa A, Tesfay SZ. Changes in biochemistry of fresh produce in response to ozone postharvest treatment. Scientia Horticulturae. 2020; (269) 109397. doi: <https://doi.org/10.1016/j.scienta.2020.109397>.

17.	Tian W, Lv Y, Cao J, Jiang W. Retention of iceberg lettuce quality by low temperature storage and postharvest application of 1-methylcyclopropene or gibberellic acid. Journal of Food Science and Technology. 2014; (51) 943-9. doi: <https://doi.org/10.1007/s13197-011-0587-6>.

18.	Zhang X, Zhang M, Xu B, Mujumdar AS, Guo Z. Light‐emitting diodes (below 700 nm): Improving the preservation of fresh foods during postharvest handling, storage, and transportation. Comprehensive Reviews in Food Science and Food Safety. 2022; (21) 106-26. doi: <https://doi.org/10.1111/1541-4337.12887>.

19.	Loi M, Liuzzi VC, Fanelli F, De Leonardis S, Creanza TM, Ancona N, et al. Effect of different light-emitting diode (LED) irradiation on the shelf life and phytonutrient content of broccoli (Brassica oleracea L. var. italica). Food Chemistry. 2019; (283) 206-14. doi: <https://doi.org/10.1016/j.foodchem.2019.01.021>

.

20.	Jin S, Ding Z, Xie J. Study of postharvest quality and antioxidant capacity of freshly cut amaranth after blue LED light treatment. Plants. 2021; (10) 1614. doi: <https://doi.org/10.3390/plants10081614>.

21.	Zhan L, Hu J, Pang L, Li Y, Shao J. Light exposure reduced browning enzyme activity and accumulated total phenols in cauliflower heads during cool storage. Postharvest Biology and Technology. 2014; (88) 17-20. doi: <https://doi.org/10.1016/j.postharvbio.2013.09.006>.

22.	Zhang Y, Xie J. The effect of red and violet light emitting diode (LED) treatments on the postharvest quality and biodiversity of fresh-cut pakchoi (Brassica rapa L. Chinensis). Food Science and Technology International. 2022; (28) 297-308. doi: <https://doi.org/10.1177/10820132211018892>.

23.	Salehinia S. Effects of different light emitting diode spectra and intensities on the stomatal functioning of lettuce and basil. 2023. doi.

24.	D'Souza C, Yuk HG, Khoo GH, Zhou W. Application of light‐emitting diodes in food production, postharvest preservation, and microbiological food safety. Comprehensive Reviews in Food Science and Food Safety. 2015; (14) 719-40. doi: <https://doi.org/10.1111/1541-4337.12155>.

25.	Nassarawa SS, Abdelshafy AM, Xu Y, Li L, Luo Z. Effect of light-emitting diodes (LEDs) on the quality of fruits and vegetables during postharvest period: A review. Food and Bioprocess Technology. 2021; (14) 388-414. doi: <https://doi.org/10.1007/s11947-020-02534-6>.

26.	Perera WPTD, Navaratne S, Wickramasinghe I. Impact of spectral composition of light from light-emitting diodes (LEDs) on postharvest quality of vegetables: A review. Postharvest Biology and Technology. 2022; (191) 111955. doi: <https://doi.org/10.1016/j.postharvbio.2022.111955>.

27.	Pennisi G, Blasioli S, Cellini A, Maia L, Crepaldi A, Braschi I, et al. Unraveling the role of red: blue LED lights on resource use efficiency and nutritional properties of indoor grown sweet basil. Frontiers in plant science. 2019; (10) 305. doi: <https://doi.org/10.3389/fpls.2019.00305>.

28.	Ngcobo BL, Bertling I. An Overview of the Recent Developments in the Postharvest Application of Light-Emitting Diodes (LEDs) in Horticulture. New Advances in Postharvest Technology. 2023. doi: 10.5772/intechopen.109764.

29.	Dhakal R, Baek K-H. Short period irradiation of single blue wavelength light extends the storage period of mature green tomatoes. Postharvest biology and Technology. 2014; (90) 73-7. doi: <https://doi.org/10.1016/j.postharvbio.2013.12.007>.

30.	Xu F, Cao S, Shi L, Chen W, Su X, Yang Z. Blue light irradiation affects anthocyanin content and enzyme activities involved in postharvest strawberry fruit. Journal of agricultural and food chemistry. 2014; (62) 4778-83. doi: <https://doi.org/10.1021/jf501120u>.

31.	Kokalj D, Zlatić E, Cigić B, Vidrih R. Postharvest light-emitting diode irradiation of sweet cherries (Prunus avium L.) promotes accumulation of anthocyanins. Postharvest Biology and Technology. 2019; (148) 192-9. doi: <https://doi.org/10.1016/j.postharvbio.2018.11.011>.

32.	Aalifar M, Aliniaeifard S, Arab M, Zare Mehrjerdi M, Dianati Daylami S, Serek M, et al. Blue light improves vase life of carnation cut flowers through its effect on the antioxidant defense system. Frontiers in Plant Science. 2020; (11) 511. doi: <https://doi.org/10.3389/fpls.2020.00511>.

33.	Aliniaeifard S, Falahi Z, Dianati Daylami S, Li T, Woltering E. Postharvest spectral light composition affects chilling injury in anthurium cut flowers. Frontiers in Plant Science. 2020; (11) 846. doi: <https://doi.org/10.3389/fpls.2020.00846>.

34.	Amagai Y, Lu N, Hayashi E, Takagaki M, Kikuchi M, Ibaraki Y, Kozai T. External green light as a new tool to change colors and nutritional components of inner leaves of head cabbages. Journal of Food Measurement and Characterization. 2021; 1-12. doi: <https://doi.org/10.1007/s11694-021-01150-y>.

35.	Costa L, Montano YM, Carrión C, Rolny N, Guiamet JJ. Application of low intensity light pulses to delay postharvest senescence of Ocimum basilicum leaves. Postharvest biology and technology. 2013; (86) 181-91. doi: <https://doi.org/10.1016/j.postharvbio.2013.06.017>.

36.	Lee S-H, Tewari RK, Hahn E-J, Paek K-Y. Photon flux density and light quality induce changes in growth, stomatal development, photosynthesis and transpiration of Withania somnifera (L.) Dunal. plantlets. Plant Cell, Tissue and Organ Culture. 2007; (90) 141-51. doi: <https://doi.org/10.1007/s11240-006-9191-2>.

37.	Lee YJ, Ha JY, Oh JE, Cho MS. The effect of LED irradiation on the quality of cabbage stored at a low temperature. Food Science and Biotechnology. 2014; (23) 1087-93. doi: <https://doi.org/10.1007/s10068-014-0149-6>.

38.	Lester GE, Makus DJ, Hodges DM. Relationship between fresh-packaged spinach leaves exposed to continuous light or dark and bioactive contents: effects of cultivar, leaf size, and storage duration. Journal of agricultural and food chemistry. 2010; (58) 2980-7. doi: <https://doi.org/10.1021/jf903596v>.

39.	Noichinda S, Bodhipadma K, Mahamontri C, Narongruk T, Ketsa S. Light during storage prevents loss of ascorbic acid, and increases glucose and fructose levels in Chinese kale (Brassica oleracea var. alboglabra). Postharvest biology and technology. 2007; (44) 312-5. doi: <https://doi.org/10.1016/j.postharvbio.2006.12.006>.

40.	Song Y, Qiu K, Gao J, Kuai B. Molecular and physiological analyses of the effects of red and blue LED light irradiation on postharvest senescence of pak choi. Postharvest Biology and Technology. 2020; (164) 111155. doi: <https://doi.org/10.1016/j.postharvbio.2020.111155>.

41.	Guo F, Zhou H, Hu H, Zhang Y, Ling J, Liu X, et al. Green LED irradiation promotes the quality of cabbage through delaying senescence and regulating glucosinolate metabolism. Food Quality and Safety. 2024; (8) fyad041. doi: <https://doi.org/10.1093/fqsafe/fyad041>.

42.	Jin P, Yao D, Xu F, Wang H, Zheng Y. Effect of light on quality and bioactive compounds in postharvest broccoli florets. Food chemistry. 2015; (172) 705-9. doi: <https://doi.org/10.1016/j.foodchem.2014.09.134>.

43.	Castillejo N, Martínez-Zamora L, Gómez PA, Pennisi G, Crepaldi A, Fernández JA, et al. Postharvest yellow LED lighting affects phenolics and glucosinolates biosynthesis in broccoli sprouts. Journal of Food Composition and Analysis. 2021; (103) 104101. doi: <https://doi.org/10.1016/j.jfca.2021.104101>.

44.	Pennisi G, Orsini F, Castillejo N, Gómez PA, Crepaldi A, Fernández JA, et al. Spectral composition from led lighting during storage affects nutraceuticals and safety attributes of fresh-cut red chard (Beta vulgaris) and rocket (Diplotaxis tenuifolia) leaves. Postharvest Biology and Technology. 2021; (175) 111500. doi: <https://doi.org/10.1016/j.postharvbio.2021.111500>.

45.	Castillejo N, Martínez‐Zamora L, Gómez PA, Pennisi G, Crepaldi A, Fernández JA, et al. Postharvest LED lighting: effect of r ed, b lue and f ar r ed on quality of minimally processed broccoli sprouts. Journal of the Science of Food and Agriculture. 2021; (101) 44-53. doi: <https://doi.org/10.1002/jsfa.10820>.

46.	Samuolienė G, Brazaitytė A, Sirtautas R, Viršilė A, Sakalauskaitė J, Sakalauskienė S, Duchovskis P. LED illumination affects bioactive compounds in romaine baby leaf lettuce. Journal of the Science of Food and Agriculture. 2013; (93) 3286-91. doi: <https://doi.org/10.1002/jsfa.6173>.

47.	Samuolienė G, Sirtautas R, Brazaitytė A, Duchovskis P. LED lighting and seasonality effects antioxidant properties of baby leaf lettuce. Food chemistry. 2012; (134) 1494-9. doi: <https://doi.org/10.1016/j.foodchem.2012.03.061>.

48.	Wilawan N, Ngamwonglumlert L, Devahastin S, Chiewchan N. Changes in enzyme activities and amino acids and their relations with phenolic compounds contents in okra treated by LED lights of different colors. Food and Bioprocess Technology. 2019; (12) 1945-54. doi: <https://doi.org/10.1007/s11947-019-02359-y>.

49.	Khan S, Dar AH, Shams R, Aga MB, Siddiqui MW, Mir SA, et al. Applications of ultraviolet light–emitting diode technology in horticultural produce: A systematic review and meta-analysis. Food and Bioprocess Technology. 2022; 1-11. doi: <https://doi.org/10.1007/s11947-021-02742-8>.

50.	Kim BS, Lee HO, Kim JY, Kwon KH, Cha HS, Kim JH. An effect of light emitting diode (LED) irradiation treatment on the amplification of functional components of immature strawberry. Horticulture, Environment, and Biotechnology. 2011; (52) 35-9. doi: <https://doi.org/10.1007/s13580-011-0189-2>.

51.	Zhan L, Hu J, Li Y, Pang L. Combination of light exposure and low temperature in preserving quality and extending shelf-life of fresh-cut broccoli (Brassica oleracea L.). Postharvest Biology and Technology. 2012; (72) 76-81. doi: <https://doi.org/10.1016/j.postharvbio.2012.05.001>.

52.	Ohashi-Kaneko K, Takase M, Kon N, Fujiwara K, Kurata K. Effect of light quality on growth and vegetable quality in leaf lettuce, spinach and komatsuna. Environmental Control in Biology. 2007; (45) 189-98. doi: <https://doi.org/10.2525/ecb.45.189>.

53.	Dou H, Niu G. Plant responses to light. Plant Factory: Elsevier; 2020. p. 153-66.

54.	Gitelson AA, Merzlyak MN, Chivkunova OB. Optical properties and nondestructive estimation of anthocyanin content in plant leaves¶. Photochemistry and photobiology. 2001; (74) 38-45. doi: <https://doi.org/10.1562/0031-8655(2001)0740038OPANEO2.0.CO2>.

55.	Meng X, Xing T, Wang X. The role of light in the regulation of anthocyanin accumulation in Gerbera hybrida. Plant growth regulation. 2004; (44) 243-50. doi: <https://doi.org/10.1007/s10725-004-4454-6>.

56.	Dou H, Niu G, Gu M, Masabni JG. Effects of light quality on growth and phytonutrient accumulation of herbs under controlled environments. Horticulturae. 2017; (3) 36. doi: <https://doi.org/10.3390/horticulturae3020036>.

57.	Perucka I, Olszówka K, Chilczuk B. Changes in the chlorophyll content in stored lettuce Lactuca sativa L. after pre-harvest foliar application of CaCl2. Acta Agrobotanica. 2013; (66). doi: : 10.5586/aa.2013.060.

58.	Hu L, Yang C, Zhang L, Feng J, Xi W. Effect of light-emitting diodes and ultraviolet irradiation on the soluble sugar, organic acid, and carotenoid content of postharvest sweet oranges (Citrus sinensis (L.) Osbeck). Molecules. 2019; (24) 3440. doi: <https://doi.org/10.3390/molecules24193440>.

59.	Hasperué JH, Guardianelli L, Rodoni LM, Chaves AR, Martínez GA. Continuous white–blue LED light exposition delays postharvest senescence of broccoli. LWT-Food Science and Technology. 2016; (65) 495-502. doi: <https://doi.org/10.1016/j.lwt.2015.08.041>.

60.	Hasperué JH, Rodoni LM, Guardianelli LM, Chaves AR, Martínez GA. Use of LED light for Brussels sprouts postharvest conservation. Scientia Horticulturae. 2016; (213) 281-6. doi.

61.	Kanazawa K, Hashimoto T, Yoshida S, Sungwon P, Fukuda S. Short photoirradiation induces flavonoid synthesis and increases its production in postharvest vegetables. Journal of agricultural and food chemistry. 2012; (60) 4359-68. doi: <https://doi.org/10.1021/jf300107s>.

62.	Taulavuori K, Hyöky V, Oksanen J, Taulavuori E, Julkunen-Tiitto R. Species-specific differences in synthesis of flavonoids and phenolic acids under increasing periods of enhanced blue light. Environmental and experimental botany. 2016; (121) 145-50. doi: <https://doi.org/10.1016/j.envexpbot.2015.04.002>.

63.	Agati G, Azzarello E, Pollastri S, Tattini M. Flavonoids as antioxidants in plants: location and functional significance. Plant science. 2012; (196) 67-76. doi: <https://doi.org/10.1016/j.plantsci.2012.07.014>.

64.	Fossen T, Rayyan S, Andersen ØM. Dimeric anthocyanins from strawberry (Fragaria ananassa) consisting of pelargonidin 3-glucoside covalently linked to four flavan-3-ols. Phytochemistry. 2004; (65) 1421-8. doi: <https://doi.org/10.1016/j.phytochem.2004.05.003>.

65.	Bárcena A, Bahima JV, Casajús V, Martínez G, Lauff D, Guiamet JJ, Costa L. The degradation of chloroplast components during postharvest senescence of broccoli florets is delayed by low-intensity visible light pulses. Postharvest Biology and Technology. 2020; (168) 111249. doi: <https://doi.org/10.1016/j.postharvbio.2020.111249>.

66.	Noodén LD, Schneider MJ. Light control of senescence. Plant cell death processes: Elsevier; 2004. p. 375-83.

67.	Ashrostaghi T, Aliniaeifard S, Shomali A, Azizinia S, Abbasi Koohpalekani J, Moosavi-Nezhad M, Gruda NS. Light intensity: The role player in cucumber response to cold stress. Agronomy. 2022; (12) 201. doi: <https://doi.org/10.3390/agronomy12010201>.

68.	Liu H, Fu Y, Wang M, Liu H. Green light enhances growth, photosynthetic pigments and CO2 assimilation efficiency of lettuce as revealed by ‘knock out’of the 480–560 nm spectral waveband. Photosynthetica. 2017; (55) 144-52. doi: <https://doi.org/10.1007/s11099-016-0233-7>.

69.	Zou Z, Yang J. Genomics analysis of the light-harvesting chlorophyll a/b-binding (Lhc) superfamily in cassava (Manihot esculenta Crantz). Gene. 2019; (702) 171-81. doi: <https://doi.org/10.1016/j.gene.2019.03.071>.

70.	Zhou F, Zuo J, Xu D, Gao L, Wang Q, Jiang A. Low intensity white light-emitting diodes (LED) application to delay senescence and maintain quality of postharvest pakchoi (Brassica campestris L. ssp. chinensis (L.) Makino var. communis Tsen et Lee). Scientia Horticulturae. 2020; (262) 109060. doi: <https://doi.org/10.1016/j.scienta.2019.109060>.

71.	Aiamla-or S, Kaewsuksaeng S, Shigyo M, Yamauchi N. Impact of UV-B irradiation on chlorophyll degradation and chlorophyll-degrading enzyme activities in stored broccoli (Brassica oleracea L. Italica Group) florets. Food Chemistry. 2010; (120) 645-51. doi: <https://doi.org/10.1016/j.foodchem.2009.10.056>.

72.	Witkowska IM. Factors affecting the postharvest performance of fresh-cut lettuce Wageningen University and Research; 2013.

73.	Kitajima K, Hogan KP. Increases of chlorophyll a/b ratios during acclimation of tropical woody seedlings to nitrogen limitation and high light. Plant, Cell & Environment. 2003; (26) 857-65. doi: <https://doi.org/10.1046/j.1365-3040.2003.01017.x>.

74.	Kotzabasis K, Strasser B, Navakoudis E, Senger H, Dörnemann D. The regulatory role of polyamines in structure and functioning of the photosynthetic apparatus during photoadaptation. Journal of Photochemistry and Photobiology B: Biology. 1999; (50) 45-52. doi: <https://doi.org/10.1016/S1011-1344(99)00067-6>.

75.	Yamazaki J-y, Suzuki T, Maruta E, Kamimura Y. The stoichiometry and antenna size of the two photosystems in marine green algae, Bryopsis maxima and Ulva pertusa, in relation to the light environment of their natural habitat. Journal of experimental botany. 2005; (56) 1517-23. doi: <https://doi.org/10.1093/jxb/eri147>.

76.	He R, Zhang Y, Song S, Su W, Hao Y, Liu H. UV-A and FR irradiation improves growth and nutritional properties of lettuce grown in an artificial light plant factory. Food Chemistry. 2021; (345) 128727. doi: <https://doi.org/10.1016/j.foodchem.2020.128727>.

77.	Zha L, Liu W, Zhang Y, Zhou C, Shao M. Morphological and physiological stress responses of lettuce to different intensities of continuous light. Frontiers in Plant Science. 2019; (10) 1440. doi: <https://doi.org/10.3389/fpls.2019.01440>.

78.	Zhu Y, Zhao X, Kang Y, Li Y, Zhang J, Cao G. A novel light source for post-harvest lettuce preservation based on chlorophyll absorption spectrum. Lighting Research & Technology. 2024; 14771535241231532. doi: <https://doi.org/10.1177/14771535241231532>.

79.	Lichtenthaler HK, Buschmann C. Extraction of phtosynthetic tissues: chlorophylls and carotenoids. Current protocols in food analytical chemistry. 2001; (1) F4. 2.1-F4. 2.6. doi.

80.	Giusti MM, Wrolstad RE. Characterization and measurement of anthocyanins by UV‐visible spectroscopy. Current protocols in food analytical chemistry. 2001; F1. 2.1-F. 2.13. doi: <https://doi.org/10.1002/0471142913.faf0102s00>.

81.	Ng SW, Rosli NSM, Razali RBR, Abdullah R, Yaacob JS. From waste to wealth: Significant potential of oil palm kernel-derived biochar as alternative substrate for production of red lettuce (Lactuca sativa L.) with high bioactive metabolites content in NFT hydroponic system. Sustainable Chemistry and Pharmacy. 2023; (34) 101167. doi: <https://doi.org/10.1016/j.scp.2023.101167>.

82.	Yen G-C, Hsieh C-L. Antioxidant effects of dopamine and related compounds. Bioscience, Biotechnology, and Biochemistry. 1997; (61) 1646-9. doi: <https://doi.org/10.1271/bbb.61.1646>.

83.	Kowalczyk K, Mirgos M, Bączek K, Niedzińska M, Gajewski M, editors. Effect of different growing media in hydroponic culture on the yield and biological quality of lettuce (Lactuca sativa var. capitata). VI Balkan Symposium on Vegetables and Potatoes 1142; 2014.
