# Supplementary material for: Green light enhances the phytochemical preservation of lettuce during postharvest cold storage
Source: PLoS One. 2024 Nov 15;19(11):e0311100. doi: 10.1371/journal.pone.0311100 (PMC11567635; doi:10.1371/journal.pone.0311100)
Supplement: Attachment — Submitted filename: Response to Reviewers.docx [file pone.0311100.s002.docx]

September 3, 2024

Sajid Ali
Academic Editor
PLOS ONE

**RE: Revisions to [PONE-D-24-22256]**

Dear Dr. Ali,

Please find our newly revised manuscript, Green light enhances the phytochemical preservation of lettuce during postharvest cold storage (PONE-D-24-22256), uploaded to the online submissions’ platform for your attention (tracked and clean copies). The reviewers’ comments were very helpful, and we appreciate the constructive criticism. We have addressed all comments in the point-by-point response below.

All authors confirm that this revised manuscript has not been previously published and has not been submitted to any other journal. Please do not hesitate to contact me if you have any further questions or require more information. We thank you again for your consideration and look forward to your feedback.

Respectfully,

Mark Lefsrud, PhD, P.Eng, P.Ag

Associate Professor and William Dawson Scholar

McGill University, Macdonald Campus

Bioresource Engineering, MS1-096, 21,111 Lakeshore

Ste-Anne-de-Bellevue, QC, Canada, H9X 3V9

Office:514-398-7967; Fax: 514-398-7990

[mark.lefsrud@mcgill.ca](mailto:mark.lefsrud@mcgill.ca)

**Editor:**

**1. Please ensure that your manuscript meets PLOS ONE's style requirements, including those for file naming.**

Response:

Thank you for your comment. The manuscript title page and file naming have been revised to ensure that it meets PLOS ONE's style requirements.

**2. PLOS requires an ORCID iD for the corresponding author in Editorial Manager on papers submitted after December 6th, 2016. Please ensure that you have an ORCID iD and that it is validated in Editorial Manager.**

Response:

Thank you for the information. The ORCID iD has been linked and validated in Editorial Manager by following the required steps.

**3. Thank you for stating the following financial disclosure: "This work was supported by UTechnology Corporation (Calgary, Alberta, Canada) and Natural Sciences and Engineering Research Council of Canada (NSERC #CRDPJ 524170-18)." 
Please state what role the funders took in the study.  If the funders had no role, please state: ""The funders had no role in study design, data collection and analysis, decision to publish, or preparation of the manuscript."" 
If this statement is not correct you must amend it as needed. 
Please include this amended Role of Funder statement in your cover letter; we will change the online submission form on your behalf.**

Response:

Thank you for the information. The financial disclosure has been revised.

The statement reads:

This work was funded by UTechnology Corporation (Calgary, Alberta, Canada) and Natural Sciences and Engineering Research Council of Canada (NSERC #CRDPJ 524170-18). However, the funders had no role in study design, data collection and analysis, decision to publish, or preparation of the manuscript.

**4. We note that you have indicated that there are restrictions to data sharing for this study. PLOS only allows data to be available upon request if there are legal or ethical restrictions on sharing data publicly. If there are no restrictions, please upload the minimal anonymized data set necessary to replicate your study findings to a stable, public repository and provide us with the relevant URLs, DOIs, or accession numbers.**

Response:

Thank you for the comment. We uploaded our data in data repository. Here are the doi and URL:

doi:10.5061/dryad.vx0k6dk1s

URL:<https://datadryad.org/stash/share/ngsVhWA13Txrbll92ZsrdhFgkmpA7sqCmyLnHTSUflk>

**5-We note that you have included the phrase “data not shown” in your manuscript. Unfortunately, this does not meet our data sharing requirements. PLOS does not permit references to inaccessible data. We require that authors provide all relevant data within the paper, Supporting Information files, or in an acceptable, public repository. Please add a citation to support this phrase or upload the data that corresponds with these findings to a stable repository (such as Figshare or Dryad) and provide and URLs, DOIs, or accession numbers that may be used to access these data. Or, if the data are not a core part of the research being presented in your study, we ask that you remove the phrase that refers to these data.**

Response:

Thank you for your comment. The phrase ‘ data not shown’ has been removed from the manuscript and the statement has been cited to our previous study.

Statement reads **(line 876-878) in revised manuscript with track change):**

The light intensity employed in this experiment was set at 10 µmol m^-2^ s^-1^ PPFD, based on our preliminary research [34].

6- **While revising your submission, please upload your figure files to the Preflight Analysis and Conversion Engine (PACE) digital diagnostic tool,** [**https://pacev2.apexcovantage.com/**](https://can01.safelinks.protection.outlook.com/?url=https%3A%2F%2Fpacev2.apexcovantage.com%2F&data=05%7C02%7Cshafieh.salehinia%40mcgill.ca%7Cdd8854594ed84ead46e808dcac265c0a%7Ccd31967152e74a68afa9fcf8f89f09ea%7C0%7C0%7C638574527962073398%7CUnknown%7CTWFpbGZsb3d8eyJWIjoiMC4wLjAwMDAiLCJQIjoiV2luMzIiLCJBTiI6Ik1haWwiLCJXVCI6Mn0%3D%7C0%7C%7C%7C&sdata=wnX7ONfYhJu9MI%2B3vfzgm6jbSMKzh0P0ND5cGYbrZng%3D&reserved=0)**. PACE helps ensure that figures meet PLOS requirements.**

Response:

Thank you for your comment. All figure files have been uploaded to the PACE to ensure they meet PLOS requirements.

**Reviewer #1:** The present experiment was carried out to assess the effects of Green and White LEDs on lettuce. I have some concern:-
There are already available literatures on effects of different lights on postharvest physiology of the fresh produce.

1. **In the present study physiological aspects such as respiration, weight loss, decay etc. are missing. Enzymology also did not include that is important part of postharvest physiology.**

Response

Thank you for the comment. We agree that including physiological aspects such as respiration, moisture loss, and decay in postharvest physiology are needed for a comprehensive understanding of postharvest quality. They have been addressed in a separate manuscript that is part of a parallel study. We are also currently conducting further experiments to assess enzymological factors, which will be detailed in a follow-up publication. This decision allows us to focus each paper on specific aspects of the research while ensuring that physiological, enzymological, and nutritional factors are adequately explored. We will reference this manuscript in our upcoming study to ensure that readers are aware of the broader scope of our research.

The statement below has been added to the conclusion to emphasize the importance of measuring other factors in future studies.

The statement reads **(lines 821-825) in revised manuscript with track change:**

Further investigation into how storage under green LED in the cold storage affects quality parameters such as moisture loss, sensory analysis, respiration rate, enzymology and microbiology in leafy greens is warranted, and additional work will explore different combinations of green light with modified atmosphere packaging.

2. **Why only Green and white lights be chosen. I think inclusion of blue and red LEDs may give more concrete results.**

Response

Thank you for the comment. In our previous studies, we demonstrated that green light has a unique ability to induce stomatal closure compared to red and blue light, which can significantly reduce transpiration and moisture loss in plants (Rufyikiri et al., 2024; Rufyikiri, 2018; Salehinia, 2023). Based on these findings, we selected green LEDs in this current work to gather more detailed data on how this specific light spectrum affects the nutritional quality of lettuce during postharvest storage. Limited studies have explored the effects of green LEDs on bioactive compounds such as flavonoids and anthocyanins and this study will add to this body of literature.

The introduction has been revised to address the need for explanation of the reason for choosing the green LED.

The statement reads **(line 113-116) in revised manuscript with track change:**

LEDs can positively influence the nutritional attributes of various fruits and vegetables [23-26], with most research focusing on red and blue light. However, limited studies have explored the effects of green LEDs on bioactive compounds such as flavonoids and anthocyanins, particularly in leafy greens.

The statement reads **(line 148-158) in revised manuscript with track change:**

Green wavelengths, specifically 500-560 nm, can induce stomatal closure more effectively than red and blue light, significantly reducing transpiration and moisture loss in spinach, kale, basil, and lettuce plants, thereby maintaining better visual quality [33, 34]. Despite these promising results, there is still limited understanding of which specific green spectra are most effective in preserving or enhancing the health-promoting properties of postharvest vegetables, especially anthocyanin and flavonoid. The aim of this work was to build on this foundational research by assessing the impact of two monochromatic green LED lights (500 nm and 530 nm) in comparison to white LED lighting (400-700 nm) and dark storage on the nutritional quality of lettuce heads stored at 5°C for 14 d.

3. **As the photographs show that author might have made some kind of chamber for light treatments, but chamber has been remained open from two sides. How did author create controlled condition for uniform distribution of lights.**

Response

Thank you for your comment. The entire setup (the chamber) was housed within a cold storage room with controlled conditions. The chamber was constructed with openings on both sides to allow for adequate air circulation and ensure that the environmental conditions, such as temperature and humidity, remained consistent across all shelves during the experiment. The open sides helped prevent the buildup of heat or moisture within the chamber, which could affect the uniformity of light distribution and potentially introduce variables that confound our results. We ensured that all other factors were controlled and consistent across treatments to focus solely on the impact of the light spectra under study.

4. **Introduction part is poorly described and needs to be improved. I recommend a comprehensive revision and resubmit.**

Thank you for your constructive criticism. The introduction has been completely revised and improved.

The revised Introduction is as follows:

Lettuce (*Lactuca sativa*) is a globally distributed and consumed leafy green, valued for its high water content, low-calorie count, and rich nutritional profile, including dietary fiber, minerals, vitamins, antioxidants, and phytochemicals such as carotenoids and phenolic compounds [1-4]. Maintaining lettuce quality postharvest presents substantial challenges due to its continued metabolic activity after harvest, leading to chlorophyll degradation, wilting, and decay [5-8]. These changes not only diminish the vegetable's appearance and nutritional value but lead to consumer rejection [1, 5-7]. Improving postharvest storage conditions directly benefits the economic viability of lettuce by extending its shelf life, maintaining its nutritional and visual quality for longer periods, and reducing waste. This ensures that more of the harvested crop reaches consumers in optimal condition, thereby maximizing market value and profitability.

Various methods have been employed to preserve postharvest quality, including chemical treatments, edible coatings, and modified atmosphere packaging [9-13]. While these techniques can be effective, they often come with concerns related to cost, effectiveness, practicality, and food safety [14, 15]. There is a growing interest in developing alternative methods that are both cost-effective and environmentally friendly. Among these, light-emitting diodes (LEDs) have emerged as a promising technology [16-19]. LEDs offer advantages such as precise control over light intensity and spectrum, non-toxicity, and cost efficiency[20-22].

LEDs can positively influence the nutritional attributes of various fruits and vegetables [23-26], with most research focusing on red and blue light. However, limited studies have explored the effects of green LEDs on bioactive compounds such as flavonoids and anthocyanins, particularly in leafy greens. Green LED light has been shown to influence various physiological and biochemical processes in plants, primarily through its interaction with specific photoreceptors like cryptochromes and phytochromes, which regulate numerous metabolic pathways. Unlike red and blue light, which predominantly activate photoreceptors at the surface level, green light penetrates deeper into plant tissues, thereby affecting internal photoreceptors [27, 28].

Guo, Zhou [29] demonstrated that green LED lighting at 25 μmol·m²·s⁻¹ for 12 d inhibited malondialdehyde accumulation and maintained higher chlorophyll content in cabbage (*Brassica oleracea* var*.* capitata) by regulating glucosinolate metabolism, while Jin, Yao [30] found that broccoli florets ([*Brassica oleracea*](https://www.sciencedirect.com/topics/food-science/cabbage) . ssp. *italica*) exposed to green LED light had higher sulforaphane content compared to those stored in darkness or fluorescent lighting for 2 d at 25°C. Castillejo, Martínez-Zamora [27] found that green LED increased total glucosinolate content by 34.5% although green LEDs had no impact on the total phenolic content on ready-to-eat broccoli sprouts (*Brassica oleracea* var. italica) during 15 days at 5 °C compared to traditional storage in darkness. These findings suggest that green LED light may influence biosynthetic pathways through pigment photoreceptor proteins, such as cryptochromes and phytochromes, leading to the accumulation of bioactive compounds, which are crucial for enhancing the nutritional quality of postharvest vegetables [27, 28].

Jin, Yao [30] demonstrated that green LED light (12 μmol m⁻² s⁻¹) increased total phenolic content and DPPH radical scavenging activity in broccoli florets. This effect was linked to enhanced activity of phenylalanine ammonia-lyase (PAL), a key enzyme in the phenylpropanoid pathway responsible for the synthesis of phenolic compounds. Green LED light has been shown to increase glucosinolate levels in broccoli, indicating that light quality and dosage can significantly influence phytochemical synthesis and overall nutritional quality [30].

Pennisi, Orsini [31] found that green LED light helped maintain color and pigment content in rocket leaves (*Diplotaxis tenuifolia*) during storage, suggesting that light quality, particularly green light, is a critical exogenous factor in regulating senescence. The capacity of harvested leaves to respond to light stimuli, mediated by photoreceptors, underscores the importance of proper light management to balance functional metabolism, such as maintaining chlorophylls and carotenoids, with the natural progression of leaf senescence [31, 32].

Green wavelengths, specifically 500-560 nm, can induce stomatal closure more effectively than red and blue light, significantly reducing transpiration and moisture loss in spinach, kale, basil, and lettuce plants, thereby maintaining better visual quality [33, 34]. Despite these promising results, there is still limited understanding of which specific green spectra are most effective in preserving or enhancing the health-promoting properties of postharvest vegetables, especially anthocyanin and flavonoid. The aim of this work was to build on this foundational research by assessing the impact of two monochromatic green LED lights (500 nm and 530 nm) in comparison to white LED lighting (400-700 nm) and dark storage on the nutritional quality of lettuce heads stored at 5°C for 14 d. Antioxidant activity, anthocyanins, flavonoids, chlorophylls, carotenoids, and total soluble solids were measuered to better understand how these wavelengths influenced the nutritional quality of lettuce during postharvest storage. By improving postharvest storage conditions using green LEDs, it may be possible to reduce waste and provide consumers with higher quality produce.

**Reviewer #2:** The manuscript is interested discussing about a non-chemical postharvest treatment. Some comments are here and in the attached reviewed manuscript need to be considered in order to improve the manuscript.

1. **Title should be specific for the nutritional quality.**

Response

Thank you for the comment. The title has been revised.

The title now reads:

Green light enhances the phytochemical preservation of lettuce during postharvest cold storage

2. - **The results are well-presented, however, not presented the full picture about the lettuce quality:the plant weight loss, the decay, the microbiology, the pink discoloration of lettuce leaf. Missing the sensory analysis since the treatment is increasing the phenolics and the chlorophyll contents.**

Response

Thank you for the comment. In this study, our primary focus was on the impact of green light on the nutritional quality of lettuce. we agree that other important aspects of lettuce quality, such as mass loss, decay, pink discoloration, and sensory analysis are essential for a better understanding of postharvest quality. We have addressed these in a separate manuscript that is part of a parallel study. This decision allows us to focus each paper on specific aspects of the research while ensuring that both physiological and nutritional factors are adequately explored. We will reference this upcoming manuscript in our current study to ensure that readers are aware of the broader scope of our research. This approach allows us to comprehensively evaluate lettuce quality, addressing the full range of factors you have highlighted.

The statement has been added to the conclusion to emphasize the importance of measuring other factors in future studies.

The statement reads **(lines 821-825) in revised manuscript with track change:**

Further investigation into how storage under green LED in the cold storage affects quality parameters such as moisture loss, sensory analysis, respiration rate, enzymology and microbiology in leafy greens is warranted, and additional work will explore different combinations of green light with modified atmosphere packaging.

**Reviewer #3:** In the manuscript PONE-D-24-22256, the authors investigated green light enhances the nutritional value and pigment preservation of lettuce during postharvest cold storage. It is my opinion that there is some useful information in the manuscript. However, the following points may be considered for improving the quality of this manuscript.

Abstract

1. **There is a mix of precise values and percentages, which can be confusing. Sticking to one form of data presentation might improve readability.**

Response:

Thank you for the comment. The abstract has been revised for consistency and reporting with percentage (%), however, increments are now presented in parentheses.

The statement reads **(line 77) in revised manuscript with track change:**

Lettuce stored with 500 nm and 530 nm green LEDs exhibited 1474.5 % and 1451.8 % (approximately 15.7 and 15.5 times) higher antioxidant activity, respectively, compared to dark storage.

Also, the statement reads **(line80-81) in revised manuscript with track change:**

Exposure to 530 nm green LEDs led to 128.2 % (approximately 2.28 times) higher anthocyanin content.

2. **The term "photoperiod (12 h-d) is unconventional and could be simplified to "photoperiod (12 hours per day).**

Response

Thank you for the comment. The term has been revised.

The term now reads (**line 75) in revised manuscript with track change:**

photoperiod (12 h per day).

Introduction

3. **There is some repetition, particularly around the nutritional benefits of lettuce and the challenges of postharvest storage. Condensing these sections could make the introduction more succinct.**

Response:

Thank you for the comment. We included the revised Introduction in its entirety in our response to reviewer 1. The sections on the nutritional benefits of lettuce and postharvest challenges have been condensed to avoid repetition and provide a more succinct overview in **line 96-105 in the Revised manuscript.**

4. **The introduction mentions various methods for preserving postharvest quality (e.g., chemical treatments, edible coatings), but it could benefit from a clearer focus on why LEDs, particularly green LEDs, were chosen for this study.**

Response

Thank you for the comment. In our previous studies, we have demonstrated that green light has a unique ability to induce stomatal closure compared to red and blue light, which can significantly reduce transpiration and moisture loss in plants (Rufyikiri et al., 2024; Salehinia, 2023). Based on these findings, we chose to focus on green LEDs in this study to gather more detailed data on how this specific light spectrum affects the nutritional quality of lettuce during postharvest storage. Also, limited studies have explored the effects of green LEDs on bioactive compounds such as flavonoids and anthocyanins.

We included the revised Introduction in its entirety in our response to reviewer 1. The sections related to your comments in the revised introduction are as follows:

**lines (106-116) in revised manuscript with track change.**

**lines (148-158) in revised manuscript with track change.**

5. **While the introduction cites many studies, it could better synthesize this information to highlight gaps in the current research. For instance, summarizing key findings from previous studies on LED effects on vegetables and then pointing out what remains unknown about green LEDs specifically.**

Response:

Thank you for the comment. The introduction has been revised to add the key findings from previous studies on green LED effects on vegetables and then pointing out what remains unknown about green LEDs specifically. We included the revised Introduction in its entirety in our response to reviewer 1. The section related to your comments in the revised introduction is as follow: **lines (113-162) in revised manuscript with track change.**

6. **The introduction mentions the economic importance of lettuce but doesn't link this directly to the benefits of improved postharvest storage. Making this connection explicit would strengthen the introduction.**

Response:

Thank you for your comment. The introduction has been revised to make a connection between economic importance of lettuce and benefits of improved postharvest storage.

We included the revised Introduction in its entirety in our response to reviewer 1. The section related to your comments in the revised introduction is as follow: **lines (96-105) in revised manuscript with track change.**

7. What specific mechanisms are proposed for the effects of green LED light on bioactive compounds? More detail on the proposed biological processes would be useful.

Response:

Thank you for the comment. While beyond the scope of this study, it is an excellent idea and we would like to explore this in future research. The introduction has been revised to include a brief explanation of the studies and their proposed biological processes through which green LED light might affect bioactive compounds.

We included the revised Introduction in its entirety in our response to reviewer 1. The section related to your comments in the revised introduction is as follow: **lines (113-162) in revised manuscript with track change.**

Results

8. **There is** **a** **significant decrease in anthocyanin content for all treatments. However,** **there is a lack of explanation for why the 530 nm green LED significantly prevented this decrease while 500 nm did not**.

Response

Thank you for the comment. The revised discussion now considers protective pigments (anthocyanin), with some added sentences expanded to include observed effects of the 530 nm green LED on anthocyanin content and possible explanations for this based on precedent literature.

The statement reads **(line 567-595) in revised manuscript with track change:**

Data presented herein show that 530 nm green LEDs significantly mitigated the loss of anthocyanin content compared to dark storage, emphasizing its potential significance in preserving these vital compounds. This effect may be attributed to anthocyanins' absorption efficiency in the blue to green spectrum, particularly around 500-550 nm [29]. Gitelson, Merzlyak and Chivkunova [42] observed that the absorbance of maple (*Acer platanoides*) and cotoneaster (*Cotoneaster alaunica*) leaves at approximately 550 nm and within the 400–600 nm band is directly proportional to anthocyanin content. Higher anthocyanin levels result in increased absorbance within this green spectral range [37]. Also, 530 nm wavelength might closely align with the optimal absorption range of anthocyanins, potentially activating protective mechanisms or biosynthetic pathways that stabilize these compounds during storage. This specific wavelength might enhance the activity of key enzymes involved in the anthocyanin biosynthetic pathway, such as chalcone synthase (CHS) or dihydroflavonol 4-reductase (DFR), leading to higher retention of anthocyanins [42, 43]. Although the effect of green light at 500 nm was relatively modest, showing similar anthocyanin content to dark conditions, it still had a positive influence when compared to white light. Red light exposure during growth significantly boosts the anthocyanin content in lettuce compared to white light treatment [44], and green and blue LEDs tend to enhance anthocyanin content in herbs [39, 45]. The implications of these findings underscore the intricate relationship between light quality and preservation of essential compounds such as plant anthocyanins, highlighting potential pathways for optimizing their accumulation through various metabolic responses [43, 46].

9. **The percentage increases and decreases are clearly reported, but the initial values again are missing, which makes it harder to interpret the results fully.**

Response

Thank you for the comment. The initial values are presented in the graphs with gray columns and they have been added to the text for the clarity.

The statement reads **(line 395-397) in the revised manuscript with track change:**

The 530 nm green LEDs prevented a considerable decrease in anthocyanin content, exhibiting only a 36.4 % decline compared to its initial value (0.53 mg/g dry matter).

10. **The initial values of total phenolic content are not provided, making it difficult to understand the magnitude of changes. Including these initial values would give a better context.**

Response

Thank you for the comment. The initial values are presented in the graphs with gray columns, and initial values have been added to the text for the clarity.

The statement reads **(line 377-379)** **in the revised manuscript with track change:**

The total phenolic content of lettuce stored in the dark and under white LEDs decreased to similar levels after 14-d storage, compared to their initial values (0.05743 g/g dry matter- p < 0.01).

Discussion

11. **The PCA and correlation matrix results are presented with a focus on clustering patterns, but the interpretation of these patterns is minimal.** **Explaining why certain clusters form and what this indicates about the biochemical changes in the lettuce would be beneficial.**

Response:

Thank you for the comment. Our interpretation of the PCA and correlation matrix data has been expanded to include the clustering patterns observed, providing a better analysis of certain clusters form and what they indicate about the biochemical changes in lettuce under different light treatments. We emphasized the strong positive association between the chlorophyll a/b ratio and the levels of flavonoids and phenolics, suggesting a potential link between pigmentation dynamics and secondary metabolite accumulation. Additionally, we highlighted the significance of the strong negative correlation between chlorophyll a and chlorophyll b, pointing to the synchronized regulation of photosynthetic pigments.

The statement reads **(lines 747-807) in the revised manuscript with track change:**

. PCA revealed distinct clustering patterns that not only differentiate the biochemical states of lettuce subjected to various light treatments but also highlight the profound impact of specific green wavelengths (500 nm and 530 nm) on metabolic processes. The clear separation of clusters corresponding to these green LED treatments from those associated with dark and white LED treatments suggests a fundamentally different metabolic trajectory. This divergence is likely driven by the selective activation of photoreceptors that are responsive to green light, which in turn modulates key biochemical pathways involved in pigment preservation and secondary metabolite synthesis [52, 64, 66].

The clustering of samples under green LED treatments can be interpreted as a manifestation of enhanced metabolic stability. Green light, particularly at 530 nm, appears to effectively modulate the chlorophyll degradation pathway, which is typically accelerated during postharvest senescence. By stabilizing chlorophyll a, which is more prone to degradation compared to chlorophyll b, the 530 nm green light helps maintain photosynthetic capacity, even under storage conditions [47, 59, 61]. This retention of chlorophyll a not only preserves the photosynthetic machinery; it additionally promotes the continued synthesis of secondary metabolites, such as flavonoids and phenolics, which are crucial for mitigating oxidative stress [67, 68]. The strong positive correlations between the chlorophyll a/b ratio and the levels of flavonoids and phenolics, as revealed by the correlation matrix, suggest a coordinated regulation of these compounds under green light. The chlorophyll a/b ratio is an indicator of the balance between light-harvesting complexes and the core photosynthetic reaction centers. A higher ratio, particularly under green LED treatments, indicates a preferential retention or synthesis of chlorophyll a, which is more efficient in capturing and converting light energy [1, 66, 69]. This enhanced photosynthetic efficiency likely provides the energy required for the biosynthesis of flavonoids and phenolics, which serve dual roles as antioxidants and photoprotective agents.

The specific wavelengths of green light (500 nm and 530 nm) appear to activate distinct sets of photoreceptors, such as cryptochromes that, are known to regulate the expression of genes involved in flavonoid biosynthesis. The upregulation of enzymes like chalcone synthase (CHS) and dihydroflavonol 4-reductase (DFR) under green light can lead to increased flavonoid accumulation, as observed in the positive correlations with chlorophyll a/b ratios [52, 53]. This synergistic effect between pigment retention and secondary metabolite production highlights the potential of green light to enhance the nutritional quality of lettuce during storage [29].

The negative correlation between chlorophyll a and chlorophyll b suggests a finely tuned regulatory mechanism that balances the degradation and synthesis of these pigments in response to green light. The selective preservation of chlorophyll a under 530 nm green LED treatment might be linked to its role in maintaining the integrity of the photosystem II reaction center, which is critical for sustaining photosynthetic activity during stress conditions, such as prolonged storage. The differential response of chlorophyll b, which is more closely associated with light-harvesting complexes, may reflect a shift in the allocation of resources towards maintaining core photosynthetic functions [47].

The PCA analysis revealed distinct clustering patterns, particularly for the 530 nm green LED treatment, indicating a unique biochemical state induced by this wavelength. This suggests that 530 nm green light not only slows the degradation of key pigments and metabolites but also promotes a metabolic state enriched in bioactive compounds. This may result from the activation of specific stress-responsive pathways, representing an adaptive response that optimizes the lettuce’s biochemical composition for extended storage. These findings underscore the potential benefits of utilizing green 500 nm and 530 nm LED light as a tool to maintain and improve the nutritional composition of stored lettuce, driving advancement of post-harvest storage systems, with the potential to bring about changes in the practices within the agricultural and food industries. These data support previous findings, which showed the effectiveness of green wavelengths in keeping stomata in a closed state in both basil (*Ocimum basilicum*) and lettuce, suggesting its potential in maintaining postharvest quality and prolonging the shelf life of leafy vegetables [34].
